# Chatbots in the fight against the COVID-19 pandemic

**DOI:** 10.1038/s41746-020-0280-0

**Published:** 2020-05-04

**Authors:** Adam S. Miner, Liliana Laranjo, A. Baki Kocaballi

**Affiliations:** 1 Department of Psychiatry and Behavioral Sciences, Stanford University, Palo Alto, CA USA; 2 Center for Biomedical Informatics Research, Stanford University, Palo Alto, CA USA; 3 Australian Institute of Health Innovation, Macquarie University, Sydney, NSW Australia; 4 Faculty of Engineering & IT, University of Technology, Sydney, NSW Australia

**Keywords:** Epidemiology, Population screening

## Abstract

We are all together in a fight against the COVID-19 pandemic. Chatbots, if effectively designed and deployed, could help us by sharing up-to-date information quickly, encouraging desired health impacting behaviors, and lessening the psychological damage caused by fear and isolation. Despite this potential, the risk of amplifying misinformation and the lack of prior effectiveness research is cause for concern. Immediate collaborations between healthcare workers, companies, academics and governments are merited and may aid future pandemic preparedness efforts.

## Introduction

**Fig. 1:**
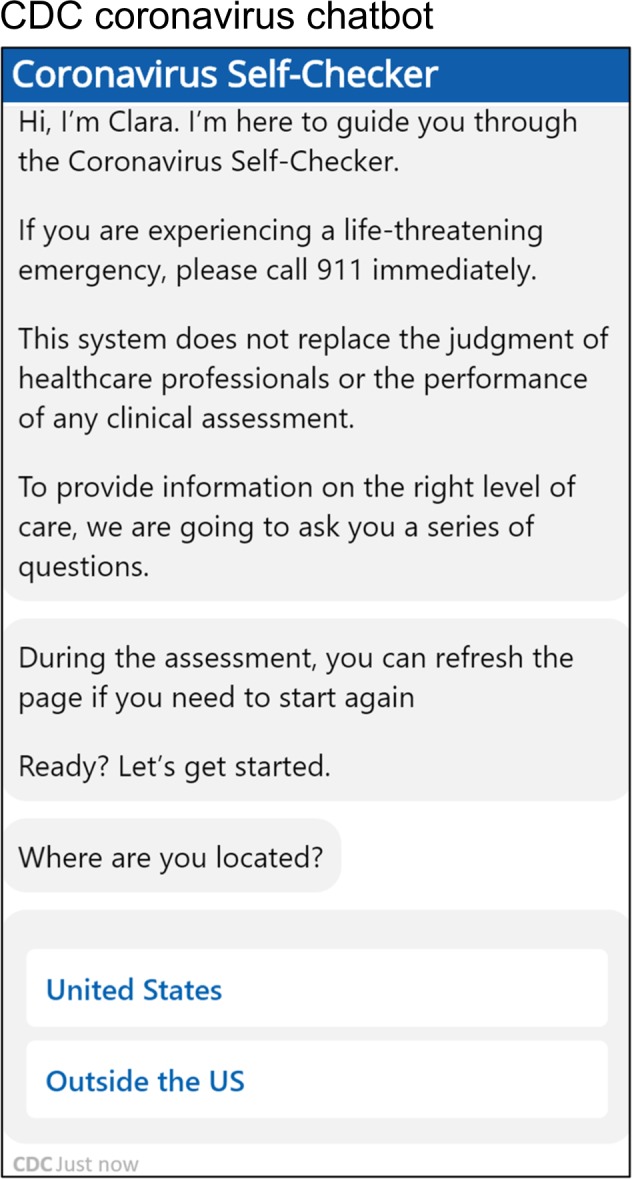
Coronavirus chatbot user interface. Chatbot publicly available at Centers for Disease Control and Prevention (CDC) website: https://www.cdc.gov/coronavirus/2019-ncov/symptoms-testing/testing.html (Captured 13 Apr 2020).

During the novel coronavirus (COVID-19) pandemic, institutions like the Centers for Disease Control and Prevention (CDC) and the World Health Organization (WHO) have begun utilizing chatbots to share information, suggest behavior, and offer emotional support^1,2^. The CDC has named theirs “Clara” (Fig. 1). Chatbots are software programs that talk with people through voice or text in their natural language^3,4^. Some well-known examples include “Alexa” from Amazon, “Siri” from Apple, and “Cortana” from Microsoft. They often come pre-installed on smartphones or home-based smart speakers^5^. In recent years, chatbot use for health-related purposes has increased considerably, from supporting clinicians with clinical interviews and diagnosis to aiding consumers in self-managing chronic conditions^6^. While promising, the use of chatbots may pose safety risks. Chatbots have varied widely in their responses to questions about physical health, suicide, intimate partner violence, substance abuse, and other sensitive conversations^4,6–9^. In one study, about a third (29%) of chatbot responses to health questions could have caused harm, and about half of those (16%) could have resulted in death if acted upon^9^. The COVID-19 pandemic puts in stark relief the potential for chatbots to help save lives.

## Challenges posed by pandemics

On 11 March 2020, the WHO Director-General “rang the alarm bell loud and clear” by calling COVID-19 a pandemic^10^. Globally and locally, control and prevention measures have been frustrated by myriad challenges. First, accurate information is crucial, but often unknown, or obscured by misinformation^11^. Second, disease fear and confusion contribute to under-reporting of symptoms^12^. Third, preventative strategies such as hand washing or social distancing are costly to disseminate and enforce. Fourth, infection countermeasures (e.g., social distancing and quarantine) are psychologically damaging^13^. For example, the SARS outbreak in 2003 resulted in a “mental health catastrophe,” in which 59% of patients in a hospitalized cohort developed a diagnosable psychiatric disorder, most commonly post-traumatic stress disorder and depression. After 30 months, less than half of this cohort psychologically recovered^14^. In this light, the WHO has called for “large-scale implementation of high-quality, non-pharmaceutical public health measures (p. 20)” to help limit new cases, and safely triage those who may be infected^15^. Normally, resources such as clinician attention or emergency department waiting areas are used at a rate the healthcare system can handle. In a pandemic, the cost of these resources being spent inefficiently or contaminated can be catastrophic.

## Special features of pandemics

Pandemics have unique characteristics that make them amenable to tailored interventions deliverable via chatbots. In particular, pandemics differ from other natural disasters in three key ways. First, individual actions can significantly worsen outcomes in a pandemic, given that a single person may infect many others depending on their behavior. Second, the fear of infecting others, especially loved ones or healthcare workers, makes infectious diseases more insidious through disease-related stigma. As a result, people can feel personally responsible for bad outcomes during a pandemic and also hide symptoms from others^12^. Third, the physical gatherings typically used to connect with others in difficult times (e.g., family meals, community centers, sports, spiritual and religious events) are exactly what we are supposed to avoid during a pandemic, worsening the risk for future mental health problems. Chatbots have unique affordances, outlined below, which may mitigate short- and long- term disease burden during infectious disease pandemics.

### Information dissemination

During a pandemic, people do not know what to do. Doing too little (e.g., not following prophylactic measures) can increase everyone’s risk of infection. Doing too much (e.g., going to the emergency room for mild symptoms) can overburden the healthcare system, wasting precious resources. Thus, reliable information sources are crucial to prevent a “misinfodemic”: the spread of a disease facilitated by viral misinformation^16^. For instance, during the Zika outbreak in 2016, misleading posts spread faster and were more popular than accurate posts on the large social-media site, Facebook^17^. Because chatbots provide a single answer to most questions, they are able to present concise information from credible sources, which may be less overwhelming than social media or web search engines’ long list of results. This matters because false news spreads online both faster and further than accurate news^18^. Chatbots, in contrast to newspapers and online information sources, can often hear and respond in natural language, improving access for people who cannot read or have difficulty using the internet. They can be available any time of the day to answer questions with up-to-date information, and unlike human experts, can concurrently speak with millions of people at the same time in local languages and dialects.

### Symptom monitoring

During a pandemic, both individuals and institutions want to know how and where infections are spreading. Individuals want to avoid getting sick, and institutions such as hospitals or local governments need data-informed policies to increase capacity (i.e., ordering more testing kits) and to plan social interventions (e.g., closing businesses). However, efforts to quickly and accurately gather population level infection rates are stymied by individuals’ fear that disclosing symptoms may harm their professional and social lives^12^. Chatbots may be uniquely well suited for symptom screening in a pandemic because people with stigmatized conditions often avoid seeking health care and education^19^. Prior research suggests people are more willing to disclose sensitive personal symptom information to a chatbot than to a human^3^. This means that people may be more forthcoming with chatbots than other humans, providing timelier and more accurate personal triage and population-level infection rate estimates. Healthcare organizations, large corporations like Apple, Amazon, Facebook, Microsoft, and Tencent, governmental agencies like the CDC, and non-governmental organizations like the WHO have launched or helped develop COVID-19 focused chatbots on platforms available to billions of users, likely with the aim of increasing accessibility^1,20,21^.

### Behavior change support

The WHO Director-General could not say it loudly enough: “all countries can still change the course of this pandemic,” and must do so by mobilizing people in transmission-reducing behaviors such as hand washing and social distancing^10^. To affect behavior, information must be actionable. Chatbots could fill the gap between knowledge and action through repetition, step-by-step instructions, and by suggesting “tips and tricks” for behavior change (e.g., self-enactable behavior change techniques)^22^. In a study of low health literacy patients in a hospital setting, 60% requested additional health information from a chatbot at discharge^23^. In a pandemic, chatbots could offload time-consuming but important behavioral support and instruction from human healthcare workers. Home-based chatbots, like the ones on devices from Amazon, Apple, and Google, may support behavior change by linking users to third-party voice apps through “skills” or “actions.” These additional capabilities allow chatbots to provide services and share information beyond their native programming.

### Mental health support

Although global and national health bodies highlight the importance of mental health in a pandemic, COVID-19 mental health needs have reportedly been under-addressed^24^. Front-line clinicians are often not trained in emergency psychological support, and mental health practitioners are in short supply^24^. Short-term, chatbots may mitigate the psychological harm of isolation, even though they cannot maintain human-level conversations. Simply disclosing concerns and receiving emotionally supportive responses can have positive value in some contexts^25^. If effectively designed and deployed, chatbots may lessen the long-term harm of pandemic-related isolation, trauma, and depression^13,26^. Preliminary evidence shows that chatbots may reduce mental health symptoms, but long-term outcomes are unclear and worthy of future investigation^6,27^.

## Challenges

Chatbots may be uniquely useful in a pandemic, but challenges in information dissemination, symptom monitoring, behavior change, and mental health support are worthy of attention. Providing reliable evidence-based information is critical in a pandemic and two issues have material impact: conflicting advice between global and local authorities, and misinformation^18^. Chatbot developers must decide whose voice to amplify and should provide reliable information from global sources like the WHO, while also coordinating with regional authorities. Both a feature and a challenge of chatbots is their ability to link users to third-party services (e.g., “skills”) that then gather and share data with unknown or unexpected consequences. If deployed for symptom screening, which is currently happening for COVID-19, constitutional and regulatory boundaries are tested by sharing health-related information between companies and governments^27,28^. This concern is not theoretical, as both the United States and Israel have reportedly explored using digital contact tracing to understand infection vectors^29,30^. Finally, although chatbots have demonstrated feasibility in behavior change and mental health treatment, they are untested in pandemics and have demonstrated limits in health crisis detection and response^4,6–9^.

These challenges, if only addressed in real time during a crisis, may lead to erroneous outputs from a lack of testing. With more than a billion voice searches per month, any health-related mistakes, such as misidentifying key symptoms, would be amplified with extensive harmful repercussions^4,9^. Additionally, medical and public health experts must inform what chatbots say, and how they say it. Translating medical information into advice for the public requires expertise and evaluation to prevent unintended consequences. Without proper design and deployment, and ongoing monitoring, chatbots may confuse rather than help users.

## Conclusion

The WHO Director-General recently called for innovative pandemic responses^10^. To this aim, chatbots are already being deployed in the fight against COVID-19^1,2,20^. If designed effectively, chatbots may help prevent misinformation, aid in symptom detection, engender infection-limiting behaviors, and lessen the mental health burden of pandemic response. In a pandemic, no group of people remains unaffected for long. Together, patients, healthcare workers, academics, technology companies, NGOs, and governments can ensure chatbots say the right thing.
